# Iron‐Based Materials for Advanced Lithium/Sodium‐Ion Batteries

**DOI:** 10.1002/cssc.202502590

**Published:** 2026-02-18

**Authors:** Jianfeng Hou, Xihan Tan, Honglei Zhang, Ning Han, Lei Jiang, Dechao Chen

**Affiliations:** ^1^ Department of Chemical and Material Engineering Lyuliang University Lvliang China; ^2^ Nottingham Ningbo China Beacons of Excellence Research and Innovation Institute Department of Chemical and Environmental Engineering The University of Nottingham Ningbo China Ningbo China; ^3^ The Edward S. Rogers Department of Electrical and Computer Engineering University of Toronto Ontario Canada; ^4^ College of Chemistry and Chemical Engineering Shanghai University of Engineering Science Shanghai China; ^5^ Quantum and Advanced Technologies Research Institute School of Environment and Science Griffith University Nathan Australia

**Keywords:** anode, cathode, iron‐materials, lithium ion battery, sodium‐ion batteries

## Abstract

Lithium‐ion batteries (LIBs) and sodium‐ion batteries (SIBs) help meet the growing global demand for sustainable energy storage due to their high energy density, portability, and rechargeability. As a key component of secondary battery systems, the anode material largely determines their overall performance. However, commercial graphite is limited by its low theoretical capacity (372 mAh·g^−1^) and poor Na^+^ storage capacity, necessitating the exploration of alternative anode materials. Among the numerous candidate materials, iron‐based compounds (including oxides, sulfides, and porous materials derived from metal–organic frameworks (MOFs)) stand out due to their high theoretical specific capacity, natural abundance, and environmental friendliness. However, severe volume expansion and structural instability during repeated charge–discharge cycles lead to rapid capacity decay, severely hindering their practical application. This review systematically summarizes the recent progress in iron‐based compounds as anodes for LIBs and SIBs. The electrochemical properties of iron oxides, iron sulfides, and porous iron‐based derivatives are highlighted, with particular attention paid to the challenges posed by volume expansion. Furthermore, a comprehensive analysis of the strategies developed to mitigate volume expansion, such as nanostructure design, carbon composites, hollow/porous structure engineering, and interface optimization, is presented. Finally, current limitations and future research opportunities are outlined, aiming to provide guidance for the rational design of high‐performance iron‐based anode materials for next‐generation rechargeable batteries.

## Graphical abstract

This Review examines iron‐based anode materials for lithium‐ and sodium‐ion batteries, emphasizing how phase chemistry (sulfides, oxides, and metal–organic framework (MOF)‐derived compounds), multiscale structural engineering, and interface regulation collectively address volume expansion, conductivity limitations, and cycling instability, while highlighting emerging roles of density functional theory (DFT)‐guided design and thermal safety considerations for practical energy storage systems.

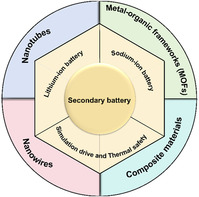

## 1 Introduction

With the continued growth of global energy consumption and the urgent need for renewable energy, efficient and sustainable energy storage technologies have become crucial for driving the transformation of the global energy mix [1, 2, 3, 4, 5, 6]. Lithium‐ion batteries (LIBs) and sodium‐ion batteries (SIBs), as typical secondary batteries, are considered promising candidates for next‐generation energy storage systems due to their high energy density and excellent electrochemical stability [7, 8, 9]. However, the overall performance of secondary batteries depends largely on the quality of the electrode materials, and the selection and preparation of the anode material play an important role [10, 11]. While graphite has been successfully commercialized for LIBs, its theoretical capacity is only 372 mAh·g^−1^, which is insufficient to meet current high energy demands [12]. Therefore, the development of new anode materials that combine high capacity with excellent cycling stability has become a core research focus for LIBs and SIBs [13, 14].

Among the many candidate materials, iron‐based compounds show great potential due to their abundant reserves, environmental friendliness, and high theoretical specific capacities [15], it plays an important role in the fields of energy and catalysis [16, 17, 18, 19]. Typical examples include iron oxide, iron sulfide (FeS), and iron‐based porous derivatives derived from metal–organic frameworks (MOFs) [20, 21]. These materials exhibit excellent lithium/sodium storage capacity in both LIBs and SIBs, and possess structural diversity and tunability, demonstrating the potential for scalable application [22, 23, 24]. However, iron‐based compounds generally face a bottleneck in cycling stability [7, 25, 26]. The root cause is the dramatic volume expansion during charge and discharge, which leads to structural pulverization [27, 28]. This not only destroys the integrity of the electrode but also causes failure of the conductive network and electrolyte interface, leading to rapid electrochemical capacity decay [29]. This dilemma has become a key challenge limiting the practical application of iron‐based materials, while also driving the extensive exploration of related structural regulation strategies [30].

To address these issues, this article systematically reviews the research progress of iron‐based compounds in LIB and SIB anodes. The article focuses on the electrochemical properties and application challenges of iron oxide, FeS, and iron‐based porous materials, and further analyzes the impact of volume expansion on cycling stability. Furthermore, this article summarizes various strategies proposed in recent years to mitigate volume expansion, including nanostructure manipulation, carbon‐based material composites, and interface optimization. By systematically sorting out the research progress and challenges of different iron‐based materials, we aim to provide ideas and references for their application in the new generation of high‐performance energy storage devices.

## 2 Iron‐Based Materials for LIBs

Iron‐based anode materials, including FeS, oxides, and MOFs, possess high theoretical specific capacities but also suffer from inherent problems. Poor conductivity, drastic volume changes during lithiation/delithiation, and unstable interfaces lead to rapid capacity decay.

In FeS‐based systems, the main challenges stem from approximately 200% volume expansion and the formation of insulating polysulfides. Fortunately, methods for constructing flower‐like, hollow, or box‐like structures, and coating the FeS surface with nitrogen/sulfur‐doped carbon or reduced graphene oxide (rGO) frameworks, can increase the electrode–electrolyte contact area and partially suppress polysulfide performance degradation, thereby achieving high specific capacity and long‐term cycle stability. For iron oxides (Fe_2_O_3_, Fe_3_O_4_), well‐designed nanostructures such as porous rhombic particles, nanoreactor structures, and hollow Fe_3_O_4_@C spheres can enhance the bonding between the electrolyte and the conductive matrix (LixPO_4_, carbon, MXene), effectively shortening the Li^+^ diffusion path and achieving high‐rate performance and excellent capacity retention after hundreds to thousands of cycles. Iron‐based MOFs and their derived composites further expand this design concept. Semicrystalline and defect‐rich Fe‐MOFs, MOF‐derived Fe_3_O_4_/C spheres, and bimetallic Fe–Co or Fe–Ni MOF derivatives possess abundant active sites and tunable porosity, forming a synergistic effect that achieves specific capacities close to or even exceeding theoretical values, and significantly improves cycle stability and rate performance. This chapter focuses on morphology engineering, carbon and MXene composites, and MOF‐derived/bimetallic structures, which serve as effective and universal strategies to alleviate the volume expansion and conductivity problems of iron‐based anodes, providing a promising direction for developing high‐energy, durable LIBs.

### 2.1 Iron Sulfide‐Based Compounds

FeS has attracted widespread attention as a promising alternative to graphite anodes [31, 32], given its excellent theoretical specific capacity (609 mAh·g^−1^), environmental friendliness, low cost, and natural abundance [33]. However, its electrochemical performance is severely limited by its poor conductivity, which is affected by the release of insulating persulfides during charge and discharge, and its have volume fluctuation of approximately 200% during lithium‐ion insertion/extraction [34], resulting in rapid capacity decay of FeS_2_ [35, 36]. Increasing the contact area between the electrode material and the electrolyte and effectively reducing the impact of persulfides on battery performance have become the primary research targets, as they can effectively utilize the electrode material to the greatest extent [37, 38]. Structural design has become an effective method for material modification.

**FIGURE 1:**
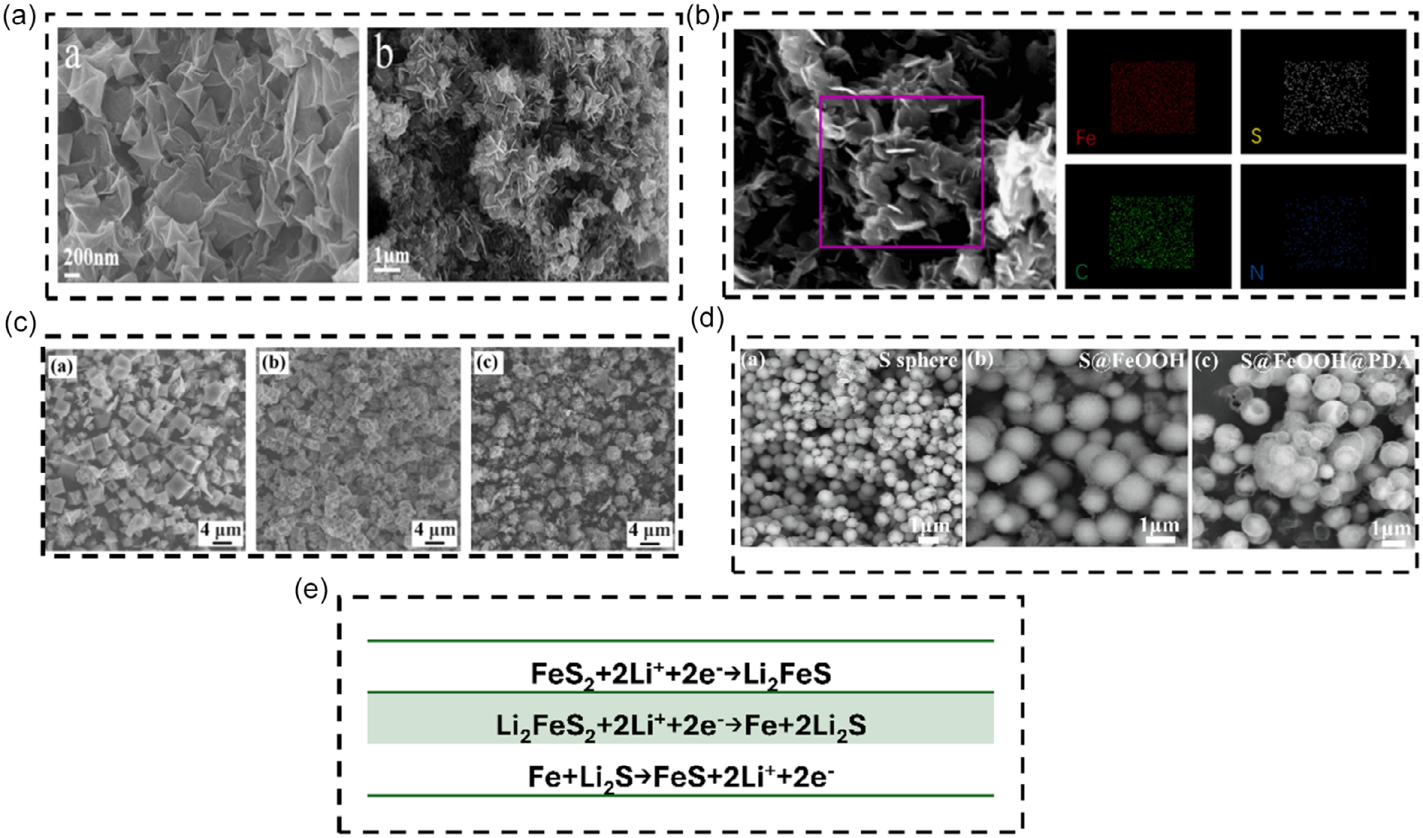
(a) SEM pictures of Fe‐MOF@GO and FeS/C@rGO. Reprinted with permission [34]. Copyright 2025, Elsevier. (b) EDS images of FeS/C@rGO. Reprinted with permission [34]. Copyright 2025, Elsevier. (c) SEM images of FeS_2_‐S, FeS–FeS_2_, and FeS@NSC composites, Reprinted with permission [39]. Copyright 2021, Elsevier. (d) SEM image of S sphere, S@FeOOH, and S@FeOOH@PDA. Reprinted with permission [40]. Copyright 2025, Elsevier. (e) Polysulfides are generated during the charge and discharge process of the FeS electrode [40].

Zeng et al. [34] prepared Fe‐based MOFs as precursors using a simple hydrothermal synthesis method. FeS was then loaded onto rGO via high‐temperature sulfurization. The resulting flower‐like FeS/C@rGO exhibited self‐assembly properties (Figure 1a). Its unique structure effectively limited the volume expansion of FeS during cycling, maintaining a reversible capacity of 1428.2 mAh·g^−1^ at a current density of 0.1 A·g^−1^ even after 130 charge and discharge cycles. In particular, the complex flower‐like structure provided an enhanced surface area, increasing the effective wetting area between the electrode material and the electrolyte, significantly shortening the lithium‐ion transport path and indirectly improving electrode conductivity. Also using a hot solvent method, Li et al. [39] prepared box‐like FeS@nitrogen‐sulfur dual‐doped carbon materials (Figure 1c) for use in LIBs. These materials exhibited a uniform particle size of 1–4 μm and a uniform distribution of F and S elements, demonstrating the material's homogeneity (Figure 1b). Furthermore, the composite material exhibits excellent long‐term stability, achieving a charge–discharge capacity of 611.8 mAh·g^−1^ over 250 cycles even at a current density of 1 A·g^−1^. This is attributed not only to the stability of the box‐shaped material but also to the fact that the polydopamine‐derived carbon coating limits volume expansion during lithium‐ion insertion and extraction. Spherical materials also facilitate increased surface area. Sun et al. [40] developed a nitrogen‐ and sulfur‐coated carbon‐coated FeS composite material, HDL‐FeS@NSC‐70 (Figure 1d). This hollow bilayer structure was prepared using an inside‐out synthesis strategy for mitigating the effects of polysulfides generated during charging and discharging (Figure 1e). The outer nitrogen and NSC improve interfacial contact with the electrolyte, further enhancing conductivity while protecting the inner FeS from damage. Consequently, a specific capacity of 558 mAh·g^−1^ was maintained even at a current density of 5 A·g^−1^. While FeS offers numerous advantages, the formation of insulating polysulfides during lithium‐ion migration is an unavoidable challenge. To address this issue, the addition of carbon‐based materials can improve conductivity to a certain extent, which is the primary reason for the improved performance of the composite materials. However, key factors determining anode stability remain the complexity of its interface structure and internal composition. Therefore, Vishwanathan et al. prepared an interface‐engineered FeS/rGO anode material. Their results showed that charge storage at high rates (e.g., 5 A·g^−1^) is primarily governed by capacitive processes, and the improved battery performance is primarily attributed to the lower diffusion barrier at the lithium‐ion diffusion interface and the presence of a built‐in electric field at the heterojunction interface [31]. Additionally, to address the high cost and difficulty of large‐scale production of chemically synthesized Fe_2_S, Meng et al. [41] successfully prepared a Fe‐xS@C/rGO electrode with a double‐layer carbon structure using natural pyrite as a raw material. This material, used in LIBs, maintained a capacity retention of 78% after 1000 cycles and exhibited a specific capacity of 356.1 mAh·g^−1^. This method provides a good reference for the high‐value utilization of natural pyrite, and as a natural material, it has been processed after reaching the end of its service life and will not cause excessive pollution to the environment.

Therefore, although FeS has the advantage of high theoretical specific capacity, the insulating polysulfides produced during the charge and discharge process are still an unavoidable problem, which will cause performance degradation and collapse of cycle stability. Therefore, the morphology engineering control and material composite of electrode materials are the main research directions [42].

### 2.2 Iron Oxide‐Based Compounds

Similar to iron‐based sulfides, iron‐based oxides possess a high theoretical specific capacity (1007 mAh·g^−1^, Equations (1)) while not generating additional impurities during charge–discharge cycles [7]. This excellent property has prompted researchers to conduct in‐depth research [43, 44, 45].

(1) $${\mathrm{Fe}}_{2}O_{3}+6{\mathrm{Li}}^{+}\to 2\mathrm{Fe}+3{\mathrm{Li}}_{2}O$$

**FIGURE 2:**
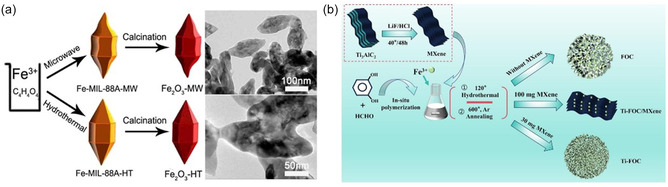
(a) Preparation Process and the SEM images of Fe_2_O_3_‐MW‐4h and Fe_2_O_3_‐HT, Reprinted with permission [46]. Copyright 2025 American Chemical Society and (b) the synthesizing process of Ti‐FOC/MXene, Ti‐FOC, and FOC composites. Reprinted with permission [47]. Copyright 2025, Elsevier.

As the most stable iron‐based oxide, *α*‐Fe_2_O_3_ is considered a promising electrode material. Currently, various Fe_2_O_3_ morphologies have been prepared and used as LIB anode materials to improve their resistance to volume expansion and electrochemical stability. Among them, nanomaterials with internal cavities are considered to have greater application prospects due to their ability to provide more reactive interfaces. To this end, Zhang et al. successfully prepared small‐scale, rhombic, porous Fe_2_O_3_ (Figure 2a) using a high‐reaction‐rate microwave‐assisted method [46]. The small size and porous nature of the Fe_2_O_3_ surface molecules facilitated full contact with the electrolyte, thereby increasing the material's capacity. Furthermore, the microwave method indirectly manipulated the Fe_2_O_3_ internal cavity structure, resulting in a high performance of 1010 mAh·g^−1^ at a current density of 1 A·g^−1^, even after 300 cycles. To address the drawback of slow ion diffusion, Dong et al. utilized a nanoreactor structure to effectively suppress coarsening of the Fe_2_O_3_ anode and accelerate lithium‐ion transfer. In this structure, Fe_2_O_3_ nanoparticle cores are uniformly distributed within an amorphous Li_*x*_PO_4_ matrix. This special structure shortens the transmission distance of lithium ions between the electrode material and the lithium source, thereby slowing down the volume expansion effect of the electrode, so that the electrode can still maintain a specific capacity of 615 mAh·g^−1^ at a current density of 20 A·g^−1^, and the capacity retention rate is close to 100% after 900 cycles at a current density of 0.5 A·g^−1^.

Similarly, Fe_3_O_4_ with the low oxidation potential (1.8 V) is considered to be a potential anode material for LIBs [48, 49]. Due to its approximately 96% expansion rate during lithiation, Wei et al. [50] prepared a nanohollow composite material. By tightly combining this material with carbon materials, the specific surface area of the active material can be increased, thereby increasing the effective reaction area. Wei et al. [50] proposed that small internal voids can cause the active material to directly burst through the outer protective layer during charge and discharge, while excessively large internal voids can result in a thin carbon shell that easily breaks. Therefore, a stable outer carbon shell is essential for ensuring the stability of the specific capacity of the electrode material during cycling. Therefore, the authors adjusted the carbon source dosage and used a template method to control the void size of the Fe_3_O_4_@C, obtaining an anode material with optimized performance and appropriate iron oxide hollowness. The specific capacity can still be maintained at 915 mAh·g^−1^ after 1100 cycles at a current density of 0.1 C, thanks to its excellent conductivity and rich surface functional groups. Therefore, Mxene has been widely used in energy storage systems such as LIBs [51]. To address the volume expansion of Fe_3_O_4_, Luo et al. [47] introduced a novel two‐dimensional material, MXene (Figure 2b), and proposed the concept of Ti doping, significantly improving conductivity. The Ti‐doped Ti‐Fe_3_O_4_/C/MXene anode exhibited significantly improved electrochemical performance compared to the pristine Fe_3_O_4_/C. At a high current density of 10 A·g^−1^, its specific capacity remained at 520 mAh·g^−1^, even after 1000 cycles. The mechanism of this improvement is attributed to the synergistic acceleration of electron transport and ion diffusion kinetics by the incorporation of Ti and MXene. This, in turn, effectively stabilized the electrode–electrolyte interface and mitigated the volume expansion of Fe_3_O_4_ during lithium‐ion insertion/extraction.

### 2.3 Fe‐Based MOF Materials

**FIGURE 3:**
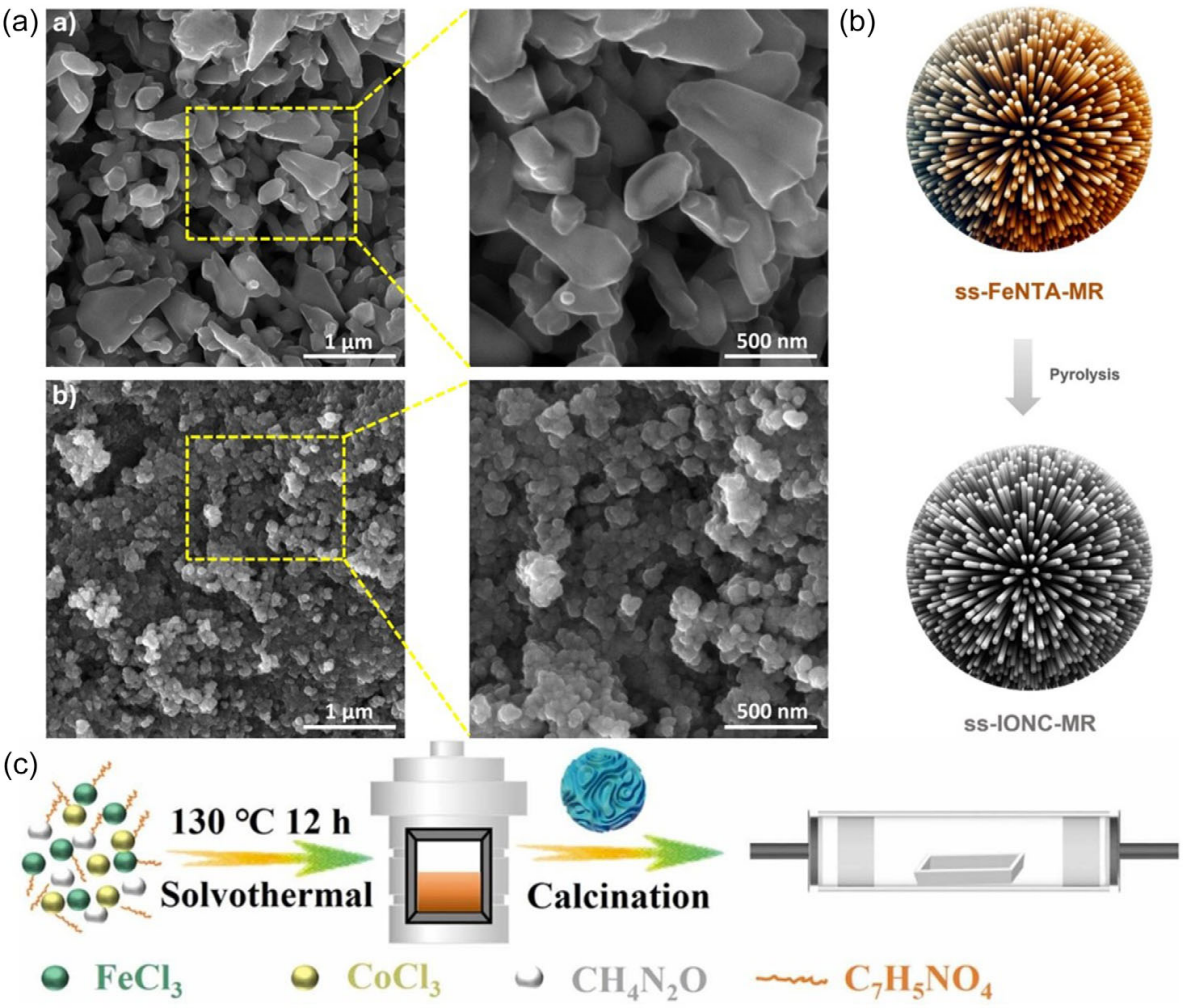
(a) SEM images of MIL‐100 (Fe) with the semi‐crystalline Fe‐MOF [54]. Copyright 2024, Elsevier. Schematic illustration of the (b) FeNTA MOF hybridization [55]. Copyright 2025, Elsevier. and (c) CoFe_2_O_4_/FeO/Fe nanocomposites produce [56]. Copyright 2022, Elsevier.

Iron‐based MOFs can significantly suppress volume expansion due to their uniquely rigid structure. Furthermore, their excellent porosity and abundant active sites make them highly attractive for LIBs [52, 53]. Moutanassim et al. [54] address the vulnerability of Fe‐based MOF (MI‐100) as an electrode material to collapse during charge and discharge, and proposed a semicrystalline strategy to enhance the electrochemical performance of MOFs. By comparing the performance of crystalline MIL‐100 (Fe), they highlighted the significant contributions of disorder and defects to LIB anode materials. The results demonstrate that semicrystalline Fe‐MOF exhibits exceptional electrochemical performance, boasting a specific capacity of approximately 854 mAh·g^−1^ and maintaining excellent cycling stability after 100 cycles at a current density of 0.1C. In comparison, the capacity of crystalline Fe‐MOF is only 195 mAh·g^−1^. Meanwhile, defective semicrystalline Fe‐MOF achieves a rate capability of up to 418 mAh·g^−1^ after 100 cycles at a current density of 2C. This exceptional performance is attributed to its unique structural features, namely its highly defective and disordered composition (Figure 3a).

Thus, iron‐based MOFs with a three‐dimensional structure rich in active sites are promising anodes for LIBs. Here, Park et al. [55] proposed a Fe‐nitrilotriacetic acid (FeNTA) MOF hybridized with melamine resin (MR) as a template to fabricate spherical structures composed of Fe_3_O_4_ carbon nanorods derived from the Fe‐based MOF. The morphology can be controlled by temperature and reaction time (Figure 3b), minimizing aggregation during cycling tests. This strategy, through radially oriented assembly of one‐dimensional MOFs or carbon nanorods, enables the facile construction of uniform three‐dimensional spherical superstructures and demonstrates potential applications in catalysis and energy storage.

Lv et al. [56] proposed an example of an iron‐based bimetallic oxide nanocomposite. By sacrificing the MOF template, they achieved additional roughness defects through a high‐temperature process while retaining the original MOF nanometer size, resulting in uniform microspheres with a diameter of 10 nm. Under the action of the bimetallic compound, the optimally formulated composite material achieved a capacity exceeding theoretical values and excellent rate capability, achieving 1120 mAh·g^−1^ even at a current density of 2 A·g^−1^ and after 500 cycles. The performance differences stem from differences in iron content, which affect lithium‐ion diffusion behavior, as verified by galvanostatic intermittent titration (GITT). Therefore, using MOFs as sacrificial templates to synthesize nanoscale iron‐cobalt‐based metal oxide composites is a simple and effective method, providing valuable insights into the design of new materials (Figure 3c). In addition to cobalt, the combination of nickel, a metal with similar chemical properties, and iron also enhances the lithium storage capacity of the composite. To this end, Dai et al. [57] prepared the heme‐like ligand TCPP (Fe) through organic synthesis and used it as an organic ligand to construct bimetallic porphyrin MOF nanofibers TCPP(Fe)‐Ni via a solvothermal method. Compared to monometallic Ni‐TCPP, this material exhibits significantly improved electrochemical performance due to the introduction of Fe^3+^ and bimetallic centers: not only does it achieve a reversible capacity of 950 mAh·g^−1^ at 0.1 A·g^−1^, but it also maintains a discharge capacity of 450 mAh·g^−1^ after 200 cycles at 1.0 A·g^−1^. This performance improvement is attributed to the increased number of active sites provided by Fe^3+^ and the synergistic effect between Fe^3+^ and porphyrin, which optimizes electronic conductivity and cycling stability. This work not only reports a high‐performance TCPP(Fe)‐Ni anode material but also provides new insights into the structural design and electrode performance optimization of bimetallic MOFs.

Hence, iron‐based materials such as FeS_2_, Fe_2_O_3_, and Fe_3_O_4_ have attracted widespread attention in the field of LIBs due to their good theoretical specific capacity. However, the volume change during the charge and discharge process leads to a decline in electrochemical performance, which greatly affects their application. Therefore, a variety of solutions have been proposed, such as morphology engineering, carbon electrode material composites, bimetallic composites, and in situ derivatization through MOF materials, all of which have achieved good results. The relevant properties and preparation methods are shown in Table 1 below.

**TABLE 1:** Summary of the performance of iron‐based materials for LIBs.

Material	Synthesis strategy	Specific capacity (mAh·g^−1^)	Current density (A·g^−1^)	Cycle	Ref.
FeS/rGO	High temperature synthesis	610	2	100	[31]
NBC/FeS	One‐pot method	972	0.1	70	[32]
FeS/NC	High temperature synthesis	728.9	1.0	600	[33]
FeS/C@rGO	High temperature synthesis	1428.2	0.1	130	[34]
R‐FeS_2_@C	Hydrometallurgy	525	0.1	100	[36]
FeS_2_/FeS/S	Ball‐milling	1044.7	0.16	30	[37]
FeS@NC	Vulcanization‐carbonization strategy	844.2	0.5	300	[38]
Box‐like FeS@nitrogen‐sulfur dual‐doped carbon	Solvothermal	611.8	1.0	250	[39]
HDL‐FeS@NSC	Inside‐out synthesis strategy	879.6	1	600	[40]
Fe1‐xS@C/rGO	Solvothermal‐melting method	356.1	1	1000	[41]
*α*‐Fe_2_O_3_/TiO_2_	One‐pot method	597.2	0.5	150	[43]
Fe_2_O_3_/MOFs	Two‐step hydrothermal	1442.7	0.1	100	[44]
CeO_2_/*γ*‐Fe_2_O_3_	Hydrothermal	456.28	0.5	100	[45]
Fe‐MIL‐88A	Microwave‐assisted‐template	1010	1	300	[46]
TMO/MXene	Hydrothermal reaction	1184	1	200	[47]
Fe_3_O_4_@C hollow	Template method	516.2	0.2	200	[50]
Semicrystalline Fe‐MOF	Room temperature synthesis	854	0.085	100	[54]
Fe_3_O_4_/N‐doped carbon	One‐pot hybridization	562.7	1.0	300	[55]

## 3 Iron‐Based Materials for SIBs

Research progress on iron‐based anode materials for SIBs focuses on FeS, Fe_2_O_3_, and MOF‐derived iron‐based composites. For FeS, morphology engineering (porous nanofibers, yolk‐shell structures, and three‐dimensional porous structures), heteroatom‐doped carbon frameworks, metal doping, and interface design (FeS/rGO) can effectively buffer volume expansion, improve conductivity, achieve high capacity, ultrafast rate performance, and long cycle life for SIBs. Fe_2_O_3_‐based anode materials can be improved through nitrogen‐doped carbon coating, self‐supporting three‐dimensional carbon fiber structures, two‐dimensional pseudocapacitive nanosheets, and amorphous Fe_2_O_3_/graphene composites. These methods collectively promote Na^+^ transport and stabilize the structure. Furthermore, MOF‐derived Fe‐based materials and heterostructures (such as Fe–N–C, FeS/MoS_2_, Fe_7_S_8_/N‐CNFs, and bimetallic Fe–Co systems) utilize porous carbon networks and engineered interfaces to achieve high reversible capacity, excellent rate performance, and ultra‐stable cycling performance, providing a multifunctional concept for advanced SIB anode design.

### 3.1 FeS for SIBs

Although LIBs, mature secondary batteries, have been commercially deployed, further development of LIBs remains limited by the shortage of lithium resources. Fortunately, sodium ions possess similar chemical properties and are more abundant, offering potential for the development of SIBs. However, while sodium resources are abundant and hold considerable market potential, their large size makes them more susceptible to electrode material collapse during charge/discharge, leading to a sharp decline in overall performance [31, 58].

Various methods have been employed to stabilize capacity fading. Yang et al. [59] proposed a strategy for tailoring the morphology of FeS anode materials, shortening the sodium ion diffusion path and improving the structure. Using simple methods including electrospinning, hydrothermal reaction, and chemical vapor deposition, they fabricated porous FeS nanofibers (FeS@NCG) coated with nitrogen‐doped carbon and rGO. This morphology‐tailored approach effectively mitigated the volume expansion of FeS during charge and discharge, resulting in excellent rate performance of approximately 300 mAh·g^−1^ at a current density of 20 A·g^−1^, even in full‐cell applications. Yuan et al. [60] also reported fabricating a unique three‐dimensional porous structure using FeS@nitrogen‐doped carbon nanosheets. This structure inhibits FeS aggregation and pulverization, enhances electron/ion conduction, immobilizes polysulfides, and increases electrolyte contact area. Furthermore, the nitrogen‐doped carbon framework provides abundant active sites for Na/K storage. As a SIB anode, this material maintains a capacity of 254 mAh·g^−1^ after 1100 cycles at 1.5 A·g^−1^; as a potassium‐ion battery anode, it maintains a capacity of 120 mAh·g^−1^ after 1100 cycles at 1 A·g^−1^, both demonstrating excellent rate performance.

**FIGURE 4:**
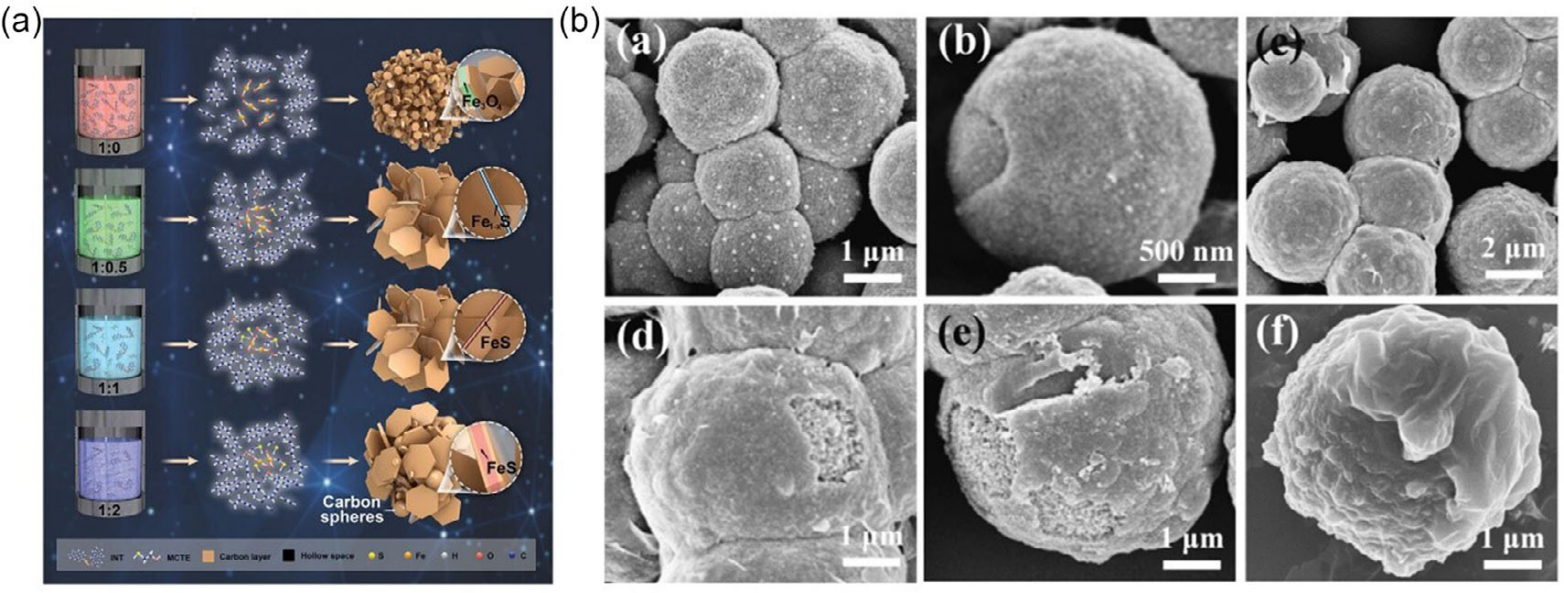
(a) Fe_3_O_4_/C, Fe1‐xS/C, FeS/C, and FeS/C@CS formation, Reprinted with permission [61]. Copyright 2024 John Wiley and Sons and (b) SEM images of FM/P‐2 with 10 (a,b) and 14 h (c,d,e,f) thermal time, Reprinted with permission [62]. Copyright 2022, Elsevier.

Importantly, uncovering the sodium ion storage mechanism of FeS‐based electrodes is crucial. To this end, Han et al. [61] proposed a one‐step vapor pressure‐induced synthesis strategy to fabricate FeS/C yolk‐shell nanosheets with hollow interstices and an ultrathin carbon layer coating. Results show that the superparamagnetic Fe nanoparticles generated during the conversion reaction induce a spin‐polarized surface capacitance effect at the Fe/Na_2_S interface, thereby storing additional ions and stabilizing ion transport (Figure 4a). This design shortens the ion transport path, mitigates volume expansion, and improves conductivity and cycling stability. The FeS/C electrode achieved a high capacity of 664.9 mAh·g^−1^ and excellent retention after 10,000 cycles in SIBs. In full cells, it exhibited a high energy density of 181.9 Wh·kg^−1^ and long‐term stability.

Metal doping can be a potential modification strategy for SIBs. Chen et al. [62] prepared Mn‐doped FeS/NC (FM/C) via a solvothermal combination with annealing. The mechanism for improved sodium ion intercalation is that the introduction of Mn atoms expands the material's lattice spacing, facilitating sodium ion insertion, thereby enhancing conductivity and rate capability. Furthermore, doping transforms FeS_2_ from a single‐core continuous growth pattern to a multicore layered growth pattern (Figure 4b), which better buffers volume changes. The PVP‐derived carbon layer further stabilizes the structure. The prepared FM/C achieved a capacity of 563.3 mAh·g^−1^ at 0.5 A·g^−1^, maintaining a capacity of 442.8 mAh·g^−1^ even at 8 A·g^−1^. Even after 8000 cycles, it retained a specific capacity of 206.2 mAh·g^−1^, and exhibited ultrafast charging capability. The assembled full cell achieved an energy density of 281.4 Wh kg^−1^, demonstrating excellent application potential. Research has shown that structural design and doping can mitigate FeS volume expansion, improve conductivity, and achieve excellent cycling and rate performance. However, improving interfacial reactions is also an effective measure to suppress volume expansion. Vishwanathan et al. [31] successfully fabricated FeS/rGO nanostructures by solid‐state annealing a FeOOH/rGO precursor at 600°C in a H_2_S atmosphere. The interfacial interaction between FeS and rGO effectively promotes electron/ion transport and reversible storage, enabling the material to maintain a stable capacity of 708 mAh·g^−1^ at high rates. At a current density of 10 A·g^−1^, its energy storage is primarily driven by capacitive processes.

### 3.2 Fe_2_O_3_ for SIBs

Fe_2_O_3_ has been extensively studied due to its abundance and high capacity, but its performance bottleneck in SIBs has prompted researchers to propose solutions from perspectives such as interfacial engineering, structural design, and energy storage mechanisms.

To address the issues of low electrical conductivity and severe volume expansion of Fe_2_O_3_ as an anode for SIBs, Song et al. [63] proposed a simple strategy using dopamine as a carbon/nitrogen source. Through self‐polymerization followed by carbonization, nitrogen‐doped graphitic carbon (NGC) layers were formed to encapsulate Fe_2_O_3_ nanoparticles. This method not only allowed control over the carbon layer thickness but also constructed a compact Fe_2_O_3_‐NGC interface, enabling a complete conversion reaction of Fe_2_O_3_ during the initial discharge process. Benefiting from this design, the Fe_2_O_3_/NGC electrode delivered a stable reversible capacity of 502.9 mAh·g^−1^ after 150 cycles at 0.1 C. Furthermore, a full cell assembled with Fe_2_O_3_/NGC as the anode and Na_3_V_2_(PO_4_)_3_ as the cathode also exhibited excellent electrochemical performance, demonstrating the potential of this strategy for practical applications in SIBs.

Similarly, Bhar et al. [64] developed a self‐supporting electrode structure based on a three‐dimensional carbon fiber network. Fe_2_O_3_ nanoparticles were synthesized via a surfactant‐assisted precipitation method and embedded into the carbon fiber substrate, forming a flexible electrode without the need for binders or metal current collectors. This design provided continuous pathways for rapid electron and ion transport while effectively buffering the volume variation of Fe_2_O_3_ during cycling. The self‐supporting electrode exhibited an excellent initial capacity (1154 mAh·g^−1^), superior cycling stability (retaining 70% capacity after 100 cycles), and a significantly enhanced Na^+^ diffusion rate (28 times faster), highlighting the remarkable advantages of this structural strategy in improving the practical performance of Fe_2_O_3_ anodes.

Vincent et al. [65] introduced a structural design of two‐dimensional Fe_2_O_3_ nanosheets, which led to pronounced pseudocapacitive behavior, endowing the electrode with fast kinetics during Na^+^ storage. The two‐dimensional morphology not only enlarged the electrode–electrolyte interface but also provided additional ion migration pathways at lattice distortions and grain boundaries, resulting in up to 95% pseudocapacitive contribution and excellent rate performance. In situ Raman analysis revealed minimal structural changes during cycling, confirming that the pseudocapacitance‐dominated mechanism can effectively avoid the structural collapse typically caused by conventional conversion reactions. This work demonstrated a facile and scalable synthesis method and proposed pseudocapacitance regulation as a new strategy for achieving high‐performance Fe_2_O_3_ anodes, offering valuable insights for the sustainable development of SIBs.

Li et al. [15] proposed an amorphous structural design strategy to improve the sodium storage performance of iron oxides. They synthesized amorphous Fe_2_O_3_/graphene nanosheet composites (Fe_2_O_3_@GNS) with an average particle diameter of around 5 nm, in which Fe_2_O_3_ nanoparticles were uniformly anchored on graphene layers via strong C‐O‐Fe bonds. Compared with crystalline Fe_2_O_3_, the amorphous composite exhibited a higher initial coulombic efficiency (81.2%), a capacity of 440 at 100, and 219 mAh·g^−1^ even at 2 A·g^−1^, all significantly outperforming the crystalline counterpart. The superior performance was attributed to the loose structure and abundant active sites of amorphous Fe_2_O_3_, along with the strong interfacial interaction with graphene, which collectively provided smoother Na^+^ diffusion channels and electron pathways while effectively buffering volume changes. This work not only validated the advantages of amorphous Fe_2_O_3_ in enhancing sodium storage performance but also provided new insights into designing amorphous structures in other transition metal oxides for optimizing electrochemical properties.

Overall, these studies not only demonstrate the diverse exploration of Fe_2_O_3_ anodes in carbon‐based composites, structural design, and sodium storage mechanism regulation, but also highlight the unique advantages of different strategies in improving capacity retention, rate performance, and cycling stability, providing important guidance for future practical‐oriented designs.

### 3.3 Fe‐Based MOF Materials

In recent years, MOF‐derived iron‐based materials have emerged as an important research direction for SIB anodes owing to their structural designability, abundant porosity, and the ability to form strong interfacial effects with heterogeneous components [66, 67, 68].

Sridhar et al. [69] proposed a rapid and efficient microwave pyrolysis method to convert DABCO‐based MOFs into Fe–N–C composites composed of iron nitride‐embedded nitrogen‐doped carbon nanotubes (Fe–N–C) grown on GO and carbon fiber substrates. Among them, MDNCNT@rGO, when used as an SIB anode, exhibited high reversible capacity and excellent cycling stability. Compared with traditional pyrolysis processes that require long‐term high‐temperature treatment under an inert atmosphere, this microwave strategy not only significantly shortened the reaction time to just a few minutes but also featured low energy consumption and high efficiency, while effectively preventing pore collapse and metal aggregation. This work not only broadened the application prospects of MOF‐derived carbon materials in energy storage and electromagnetic protection but also highlighted the advantage of using conductive substrates to induce directional growth of carbon nanotubes, providing a new design concept for high‐performance multifunctional electrode materials. Fu et al. [70] employed an iron‐based MOF (MIL‐100(Fe)) as the precursor and synthesized flower‐like FeS/MoS_2_ composites through subsequent sulfidation, combined with density functional theory (DFT) calculations to reveal that the heterostructure exhibited metallic characteristics and interfacial effects that enhanced electronic transport and structural stability. The unique FeS/MoS_2_ heterointerface could both capture polysulfide intermediates and buffer electrode volume expansion, thereby improving sodium storage performance. Electrochemical tests showed specific capacities of 464 and 365 mAh·g^−1^ at 0.1 and 1.0 A·g^−1^, respectively; even at 5 mAh·g^−1^, the composite retained 325 mAh·g^−1^ (71.1% retention), and after 200 cycles at 2.0 A·g^−1^ it still delivered 331 mAh·g^−1^. Compared with single‐component FeS or MoS_2_, the composite exhibited superior cycling stability and rate capability. This study demonstrated the feasibility of constructing two‐dimensional composite structures using Fe‐MOFs as templates, providing a new strategy for the development of high‐performance SIB anodes.

Wang et al. [71] fabricated MOF‐derived Fe_7_S_8_ nanoparticles/nitrogen‐doped carbon nanofiber composites (Fe_7_S_8_/N‐CNFs) via electrospinning combined with sulfidation. In this structure, Fe_7_S_8_ nanoparticles with an average size of −17 nm effectively shortened the Na^+^ diffusion path, while the three‐dimensional interconnected N‐doped carbon nanofiber network not only significantly improved the overall conductivity, but also mitigated nanoparticle agglomeration and volume expansion during cycling, and provided abundant active sites for Na^+^ storage as well as electrolyte diffusion channels. Benefiting from this synergistic design, the Fe_7_S_8_/N‐CNF anode exhibited excellent electrochemical performance: a reversible capacity of 649.9 mAh·g^−1^ after 100 cycles at 0.2 A·g^−1^, and after 2000 cycles at 1 A·g^−1^, it still maintained 472.1 mAh·g^−1^ with an ultralow capacity decay rate of only 0.00302% per cycle. Furthermore, a full cell assembled with Fe_7_S_8_/N‐CNFs as the anode and Na_3_V_2_(PO_4_)_3_/C as the cathode also showed outstanding energy storage properties. This synthesis strategy features simplicity, low cost, and high yield, offering a practical pathway to ultrastable, high‐performance SIB anodes.

Zhao et al. [72] used a bimetallic MOF as the precursor to prepare derived three‐dimensional conductive porous carbon nanosheets (CPCN). Specifically, Fe–Co oxide (FCO) nanowires were grown on the CPCN surface via a hydrothermal method, followed by modification with Au nanoparticles, to construct a flexible FCO/Au/CPCN@CC electrode. This structure provided excellent conductivity and a large surface area, while enhancing the dispersion and utilization of active materials. Electrochemical measurements revealed that as an anode for SIBs, FCO/Au/CPCN@CC delivered a high capacity of 958 mAh·g^−1^ at 0.1 C, and maintained coulombic efficiency above 98% even after 500 high‐rate cycles. The synergistic effect between Au nanoparticles and FCO nanowires/porous carbon nanosheets enabled outstanding performance in both batteries and supercapacitors, offering a new approach for the development of multifunctional energy storage devices.

Overall, these studies fully demonstrate the importance of structural regulation and interfacial design achieved through MOF‐derived strategies. On the one hand, rapid and efficient synthesis methods enhance the feasibility of material preparation; on the other hand, heterostructure construction and carbon network integration significantly improve conductivity and stability, thereby greatly enhancing the cycling life and rate capability of SIB anodes. These works provide valuable insights and references, as shown in Table 2 for the development of high‐performance and multifunctional energy storage materials.

**TABLE 2:** Summary of the performance of iron‐based materials for SIBs.

Synthesis strategy	Masterial	Specific capacity (mAh·g^−1^)	Current density (A·g^−1^)	Cycle	Ref.
Ice bath amorphous synthesized	Fe_2_O_3_@GNS	110	2	500	[15]
Sol‐gel method	3D FeS@N nanosheets	254	1.5	1100	[60]
Vapor‐pressure induced synthesis route	FeS/C yolk‐shell	69	0.1	10000	[61]
Solid‐state annealing method	FeS/rGO	750	0.5	150	[67]
N‐doped graphitic derived	Fe_2_O_3_/NGC	502.9	0.1	150	[63]
Carbon fiber‐based freestanding electrode architecture	Fe_2_O_3_‐C@CF	533	0.03	100	[64]
Two‐step heating process	Fe_2_O_3_‐700	100	0.5	250	[65]
Box‐like FeS@nitrogen‐sulfur dual‐doped carbon	Solvothermal	473.2	0.2	100	[39]

## 4 Density Functional Theory (DFT): Emerging Tools for Electrode Design

In recent years, DFT has been widely applied to the mechanistic studies of iron‐based anode materials, providing important theoretical support for optimizing their energy storage performance [31, 61, 73].

**FIGURE 5:**
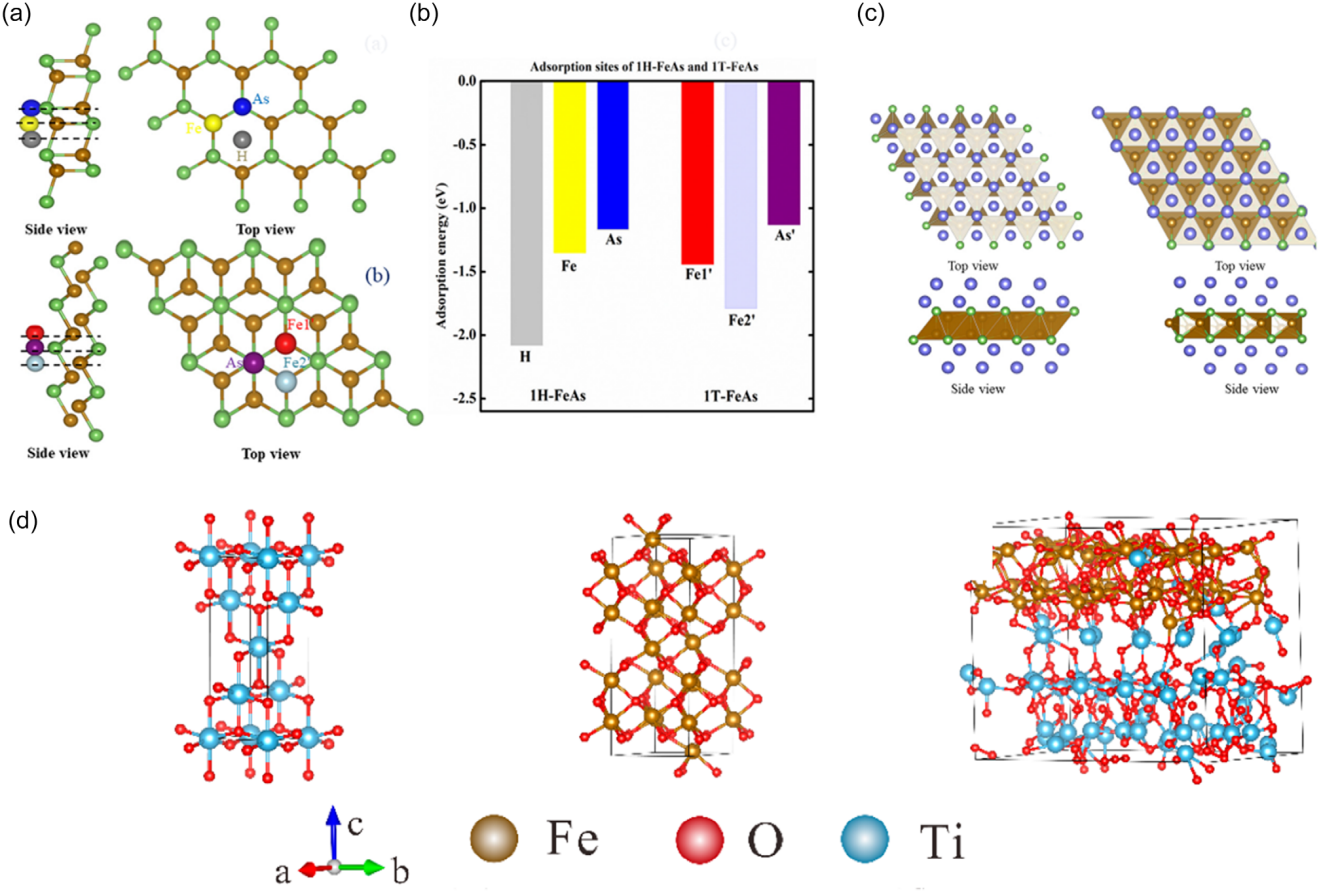
(a) Possible adsorption sites of 1H‐FeAs and 1T‐FeAs monolayers, with (b) their adsorption energies. (c) Top and side views of Li layers on 1H‐FeAs and 1T‐FeAs (Reprinted with permission [74]. Copyright 2024 Royal Society of Chemistry). (d) Optimized structures of TiO_2_, Fe_2_O_3_, TiO_2_@Fe_2_O_3_, and corresponding DOS [75].

Kumar et al. [74] systematically investigated the structural, electronic, and electrochemical properties of two hexagonal iron arsenide monolayers (1T‐FeAs and 1H‐FeAs) based on DFT calculations (Figure 5a–c). The results showed that both phases exhibited metallic character with spin‐polarized band structures and theoretical capacities of ∼374 mAh·g^−1^, higher than that of graphite. The 1T‐FeAs phase demonstrated superior ionic conductivity and a lower Li diffusion barrier (0.38 eV), corresponding to faster charge/discharge rates, with an average open‐circuit voltage of 0.44 V compared to 0.61 V for 1H‐FeAs. At maximum Li adsorption, both phases exhibited smaller volume expansion than graphite, indicating good structural stability. In addition, Li adsorption induced a transition in 1H‐FeAs from ferromagnetic to antiferromagnetic, while 1T‐FeAs remained almost unchanged. This study provided theoretical evidence for exploring the potential of Fe‐based two‐dimensional materials as LIB anodes. Chen et al. [76] evaluated the potential of FeS monolayers as anchoring materials for lithium‐sulfur batteries through first‐principles calculations. The results showed that FeS exhibited moderate adsorption energies (1.00–3.26 eV) for S_8_ and Li_2_Sn (*n* = 1–8), effectively suppressing the shuttle effect while maintaining structural stability. The adsorption of higher‐order polysulfides (Li_2_S_4_, Li_2_S_6_, Li_2_S_8_) was dominated by van der Waals interactions, whereas stronger chemical adsorption was observed for lower‐order products (Li_2_S, Li_2_S_2_) (Figure 5d).

Moreover, FeS remained metallic after adsorption and exhibited extremely low diffusion barriers (0.007–0.43 eV), facilitating rapid Li_2_S migration, preventing agglomeration, and accelerating reaction kinetics. Overall, FeS monolayers were identified as highly promising anchoring materials for lithium‐sulfur batteries. Tang et al. [77] synthesized FeS_2_/Fe_7_S_8_‐rGO composites via a solvothermal method, which exhibited excellent cycling stability and rate capability as anodes for LIBs and SIBs (e.g., retaining 514 mAh·g^−1^ after 3000 cycles at 2.0 A·g^−1^). The rGO component effectively improved conductivity and mitigated volume expansion, while electrochemical kinetics analysis revealed significant pseudocapacitive contribution. DFT calculations further demonstrated the synergistic effect of the FeS_2_/Fe_7_S_8_ heterointerface, achieving a favorable balance between Li/Na adsorption energy and diffusion barriers (reduced to 0.34/0.45 eV), thereby promoting ion transport and enhancing electrochemical performance. Chen et al. [75] prepared lychee‐like TiO_2_@Fe_2_O_3_ core–shell microspheres via a homogeneous precipitation method, which exhibited remarkable performance enhancement as LIB anodes. After 200 cycles at 0.2 C, the composite delivered a capacity of 591.5 mAh·g^−1^, representing a 219.3% improvement over TiO_2_, and retained 273.1 mAh·g^−1^ after 500 cycles at 2 C, outperforming commercial graphite. Electrochemical impedance spectroscopy revealed lower charge transfer resistance and faster Li^+^ diffusion. DFT calculations further confirmed that TiO_2_@Fe_2_O_3_ exhibited metallic states, and density of states (DOS) analysis verified that the high conductivity originated from interfacial electronic structure modification, thereby explaining its superior rate and cycling performance. This work provided both experimental and theoretical support for the development of efficient iron‐based composite anodes. Zhang et al*.* [78] addressed the issues of volume expansion, sluggish kinetics, and interfacial instability in metal sulfides by designing Sb/Fe_2_S_3_ composite anodes. Through ball milling and compounding with acetylene black (Sb/Fe_2_S_3_‐C), the composite exhibited improved conductivity and structural stability. DFT calculations predicted the possible reaction pathways between Fe and Sb_2_S_3_ through Gibbs free energy (Δ*G*) analysis, indicating that a Fe/Sb_2_S_3_ molar ratio of 2:1 favored the formation of Fe_2_S_3_‐Sb heterojunctions, thus guiding experimental synthesis. Experimental validation showed that Sb/Fe_2_S_3_‐C delivered outstanding rate performance in LIBs (capacities from 1170.6 to 345.3 mAh·g^−1^ at 0.1–10 A·g^−1^) and good cycling stability in SIBs (501.6 mAh·g^−1^, after 80 cycles). This study provided both theoretical guidance and experimental evidence for the design of Fe‐based polymetallic sulfides.

Collectively, these studies not only demonstrate unique advantages of DFT in revealing the electronic structures, ion diffusion behaviors, and interfacial effects of iron‐based materials but also emphasize the critical role of combining theoretical calculations with experimental validation in advancing high‐performance iron‐based anodes.

## 5 Thermal Safety Issues of LIBs and SIBs

With the continuous improvement of battery energy density and system integration scale, battery thermal runaway (TR) has become a key factor restricting the safe application of energy storage systems. For LIBs and SIBs, TR is no longer a simple process of “overheating and decomposition” of a single material, but a complex evolutionary behavior triggered by a series of electrochemical/chemical reactions and physical processes. TR can be described as a series of consequences, including solid electrolyte interface (SEI) decomposition, electrode/electrolyte reactions, gas evolution, internal short circuits, fire, and even explosion. The underlying mechanism that truly dominates the failure is still under investigation. Therefore, only by deeply understanding the thermal hazard mechanism at the material‐battery‐system multilevel can targeted mitigation strategies be proposed [79].

For high‐energy LIBs, the contradiction between energy density and safety has been widely confirmed: as the chemical energy stored per unit mass/volume increases, the reactivity of electrode materials significantly increases. Once entering the runaway range, heat release becomes more concentrated, the temperature rise rate is higher, and the time window is narrower, easily leading to violent fires or even explosions. Existing research indicates that the dominant heat sources of TR include not only the thermal instabilities of the positive electrode, negative electrode, and electrolyte, but more importantly, the strong thermal crosstalk occurring within their highly overlapping reaction temperature ranges. This crosstalk manifests in the interactions between battery components and is caused by harmful byproducts of side reactions [80]. Additional exothermic oxidation reactions occurring in ultra‐high temperature ranges exceeding approximately 1000°C further accelerate temperature rise. While current mitigation methods (such as nonflammable electrolytes, solid‐state or aqueous electrolytes) can reduce the combustion risk induced by carbonate solvents, they often come at the cost of energy density or introduce new safety hazards such as dendrite short circuits and new interface side reactions. Therefore, these strategies, in most cases, can only delay the triggering of TR or reduce its intensity, falling far short of fundamentally eliminating catastrophic heat release [80].

SIBs, due to the abundance and low cost of sodium resources, are considered a strong candidate for large‐scale energy storage; however, their thermal safety issues are equally significant. On the one hand, some sodium‐based cathode systems and full batteries exhibit lower self‐heating rates and total heat release compared to comparable lithium‐ion systems, demonstrating potentially safer characteristics under certain operating conditions. On the other hand, Na^+^ has a larger radius and weaker Lewis acidity, which easily leads to faster electrode structure degradation and higher SEI solubility, thus exposing new active interfaces under cycling and abuse conditions, inducing more severe exothermic side reactions. Different cathode families (layered oxides, Prussian blue analogs, polyanionic compounds, etc.) show significant differences: some materials are prone to releasing oxygen at high temperatures or reacting with organic electrolytes to produce toxic gases and large amounts of heat, raising serious concerns about their safety in large‐scale energy storage scenarios; while polyanionic cathodes, although having relatively lower specific capacity, possess excellent thermal stability and cycle durability, and are considered more suitable for energy storage applications [81]. For the anode, hard carbon remains the mainstream choice [82], but its thermal safety relative to lithium‐ion graphite in its sodium‐modified state is still inconclusive, especially in the deeply sodium‐modified state, highly active sodium clusters are generated. Once the organic SEI fails at high temperatures, the intense exothermic reaction between these sodium clusters and the electrolyte could become the dominant heat source for TR [83].

At the materials design level, increasing evidence suggests that mitigating TR requires a coordinated approach from three aspects: heat source reduction, heat storage regulation, and thermal conductivity optimization, rather than solely relying on external protection circuits or battery management systems. Interface engineering offers a promising path to “weaken the trade‐off” between high energy density and thermal safety. For example, the recent construction of a multicomponent high‐entropy inorganic SEI on the surface of a hard carbon anode demonstrates the effectiveness of this approach: the inert inorganic components stabilize the interface structure and improve the thermal stability of the SEI, providing high‐temperature protection for the deeply sodium‐modified anode; the active inorganic components construct multidimensional Na^+^ transport channels, significantly reducing the diffusion barrier and enabling the safe formation of high‐capacity sodium clusters driven by pore filling within the controlled interface [83]. Thanks to the synergistic effect of this multicomponent inorganic interface, the full cell not only achieves high‐load long‐cycle operation (e.g., maintaining approximately 80% capacity after 800 cycles under high areal load conditions, with an Ah‐level cylindrical cell energy density reaching around 146 Wh·kg^−1^), but also fully demonstrates that a reasonable interface and microstructure design can significantly suppress key exothermic channels while maintaining or even improving energy density [83].

Overall, research from Li^+^ and Na^+^ systems is gradually forming a consensus: thermal safety should be considered an active aspect of battery system design, rather than a passively imposed condition after performance optimization. Future work urgently needs to shift from evaluating the thermal stability of single components to a holistic perspective involving the multiscale coupling of materials, interfaces, the entire cell, and battery packs/systems. This requires establishing multilevel connections from thermodynamics and kinetics, interface evolution and crosstalk, individual cell thermal behavior to the module level. Simultaneously, it is necessary to develop more realistic, repeatable, and quantifiable safety evaluation methods, including controllable triggering methods, signal‐based early TR identification, and thermal safety assessment of aged batteries. Combining big data and artificial intelligence techniques for high‐throughput screening of materials and systems holds promise for guiding the development of next‐generation energy storage battery systems that combine high energy density, long lifespan, and intrinsic thermal safety [79, 80, 81, 82, 83].

## 6 Conclusion and Outlook

Iron‐based compounds, due to their abundant resources and high theoretical specific capacity, have shown great potential for application in LIBs and SIBs. However, the volume expansion during charge/discharge remains a key bottleneck hindering their large‐scale application. To address this challenge, current research has focused on nanostructure design, carbon‐based material composites, and pore structure manipulation. While this has improved cycling stability to some extent, it remains far from practical application.

Therefore, future research could focus on the following directions: (1) The composition and structure of the solid electrolyte interphase (SEI) significantly influence the volume change and interfacial stability of iron‐based compounds during charge/discharge. Therefore, in‐depth research on the formation mechanism and control strategies of the SEI will provide new insights for mitigating volume expansion and extending cycling life. (2) When combined with artificial intelligence and machine‐learning methods, first‐principles calculations can accelerate material screening and structural optimization, potentially enabling a shift from “empirical‐driven” to “mechanism‐ and data‐driven” approaches. (3) Future material development must balance environmental friendliness and resource sustainability while optimizing performance. Toxic components should be avoided, environmentally friendly compounds or complexes should be prioritized, and attention should be paid to material recyclability to promote the green application of iron‐based electrode materials. (4) Beyond laboratory‐level innovation, developing simple, low‐cost, and scalable synthesis processes is crucial. The scalability and economic viability of the preparation route will directly determine the application prospects of iron‐based compounds in practical batteries. (5) The storage and release of electrical energy in LIBs and SIBs inevitably generate heat. Recent fires and explosions related to advanced secondary batteries have prompted the search for more advanced strategies and new, safer alternatives.

In summary, future research on iron‐based compounds in LIBs and SIBs requires a comprehensive consideration of structural regulation, interface stability, theoretical guidance, and the coordinated development of green processes to truly advance their advancement from laboratory exploration to large‐scale application.

## Funding

This work was supported by the Australian Research Council (DE250100132) and the Key Research and Development projects of Lvliang City (2023GXYF09).

## Conflicts of Interest

The authors declare no conflicts of interest.
